# Freeze-Dried Chitosan Scaffolds Containing Grape Seed Oil for Wound Healing Applications

**DOI:** 10.1021/acsomega.5c06168

**Published:** 2025-10-06

**Authors:** Erik Felix dos Santos, Larissa Ribeiro Lourenço, Carlos Alberto da Silva, Juliana Marchi

**Affiliations:** † Center of Natural and Human Sciences, Federal University of ABC, Santo André, São Paulo 09280-560, Brazil; ‡ Center of Natural and Human Sciences, Federal University of ABC, São Bernardo do Campo, São Paulo 09606-045, Brazil

## Abstract

Wound healing is the ultimate goal in skin repair and tissue engineering. Skin is highly susceptible to injuries, with a mortality rate in chronic wounds comparable to cancers, thus imposing a high cost ($126.8bi/USA) to health care systems. Chronic wound regeneration stagnates in the inflammatory phase, progressing after its control. Porous biomaterials with a tunable degradation that control bleeding and exudate and maintain a moist environment could improve skin repair. Chitosan is biocompatible, biodegradable, antimicrobial, and hemostatic. *Vitis vinifera* (grape) seed oil (grape seed oil (GSO)) has a high content of unsaturated fatty acids with antioxidant and anti-inflammatory activity. In this study, 0.3% and 0.6% (v/v) GSO were incorporated into chitosan without cross-linker via freeze-drying process aiming scaffolds with enhanced properties for wound healing applications. The porous scaffolds maintained their shape throughout the 28 days in phosphate buffer solution. The overall physical and chemical characterizations showed that GSO was fully incorporated into the chitosan scaffolds (CSs), even after neutralization in NaOH, demonstrating a durable incorporation. Scanning electron microscopy images revealed superficial and interconnected pores in a stratified structure, possibly favorable to cell proliferation and nutrient transport The addition of 0.6% GSO increased porosity by 17%, without altering freeze-drying parameters, possibly by the coalescence of oil droplets during freezing. Moreover, the scaffolds showed high swelling, up to 800%, indicating the potential for exudate control. Higher GSO concentration leads to lower degradation rates in vitro, which is compatible with the wound closure time in chronic wounds. The biological in vitro results showed the high biocompatibility of GSO and the scaffolds in epithelial Vero cells. Altogether, our results show that incorporating GSO in CS using a freeze-drying process allows scaffolds with modulated morphological and physicochemical features, maintaining the biocompatibility of these natural products, making them a promising biomaterial for wound healing.

## Graphical abstract

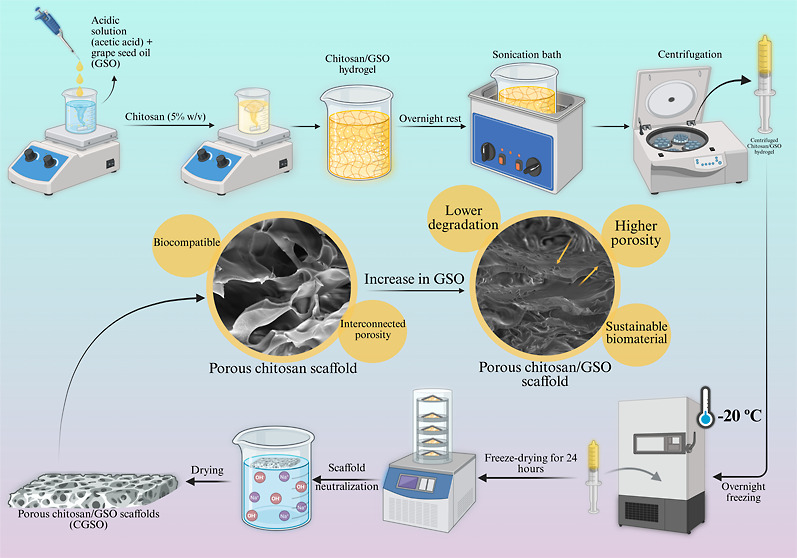

## 1 Introduction

Skin wounds are among the most common types of injuries,^1^ affecting approximately 8.2 million persons in 2024, with costs reaching up to $96.8 billion.^2^ In 2016, the costs of hospital-acquired pressure injuries were estimated at $26.8 billion, affecting approximately 2.5 million individuals.^3^ In a global perspective, the highest expenditures on wound treatment are from the United States, China, and Japan ($126.86, 26.94, and 18.89 billion, respectively),^4^ in a scenario in which mortality from chronic injuries can be comparable to some types of cancer.^5^

The skin is the largest organ in the human body, covering ∼2 m^2^ and weighing up to 15% of total body weight.^10^ The skin comprises two layers: the epidermis, formed by epithelial tissue, and the dermis, formed by connective tissue, anchored in the hypodermis. The epidermis is a keratinized stratified squamous epithelium composed of up to five layers: stratum basale, spinosum, granulosum, lucidum, and corneum. It is an avascular tissue composed of keratinocytes, melanocytes, and intraepidermal macrophages, which acts as a selective barrier in the transfer of substances, as secretory surface, and in protection against abrasive influences from the external environment, ensuring the body’s homeostasis.^11^ The dermis is composed of fibroblasts, macrophages, and elastic and collagen fibers and is thicker than the epidermis. The dermis is divided into two regions: the papillary and reticular. The hypodermis is a loose connective tissue layer that anchors the skin to internal body structures.^12^

Wounds are injuries to the skin due to external action, medical, or physiological conditions that impact the structure and function of the tissue.^6^ They can be classified according to (i) the skin layers affected: superficial (epidermal), partial-thickness (dermal), and full-thickness wounds (subcutaneous fat); (ii) regeneration: acute or chronic. Acute wounds, such as cuts or abrasions caused by mechanical injuries, usually fully regenerate within 8–12 weeks. Chronic wounds regenerate more slowly and can sometimes reappear, occurring due to predispositions such as diabetes, persistent infections, or poor primary treatment.^7^ Conventional wound treatments involve debridement of necrotic tissue, wound cleaning, use of topical antimicrobial agents, maintenance of moisture, and, in some cases, compression or the use of biological skin substitutes that act as an epidermal barrier, thus improving regeneration.^8^ Although autograft is considered the gold standard in wound treatment, there is a shortage of tissue, while there are risks of host reaction and disease transmission with allograft.^9^ An alternative that can overcome such limitations is the development of optimized scaffolds for skin regeneration.

The skin has intrinsic regenerative properties, from the keratinization process occurring in the basal epithelium^13^ to the formation of granulation tissue and scars.^14^ Acute wound regeneration occurs in four concomitant stages: hemostasis, inflammation, proliferation, and remodeling. In the inflammatory stage, macrophages eliminate bacteria and clean the surface.^15^ At this stage, monocytes differentiate into macrophages, which participate in stimulating hair growth, collagen synthesis, and fibrosis. The two main types of macrophages in the wound bed are the M1 type, with antimicrobial properties due to the release of inflammatory mediators, and the M2 type, which suppresses inflammatory reactions and adaptive immune response and favors angiogenesis.^16^ In chronic wounds, regeneration stagnates in the inflammatory stage, in which neutrophil infiltration and the presence of reactive oxygen species occur, and only continues after inflammation is controlled.^19^

Biomaterials for wound treatment must improve the natural regenerative process by ensuring the desired conditions in each phase, including factors such as allowing cell adhesion, proliferation, and differentiation; maintaining a moist environment; being permeable to water; and absorbing blood and exudate, as this causes the separation of tissue layers, delaying regeneration.^20^ Biomaterials must be biocompatible, have antimicrobial action, a degradation rate similar to the growth of new tissue, and suitable microstructural characteristics including porosity, pore size, and interconnectivity between pores.^21,22^ Several manufacturing methods for polymeric scaffolds can be used to optimize these properties, such as solution casting, electrospinning, 3D printing, and freeze-drying. The last one can be used both for synthetic and natural polymers, such as collagen, gelatin, alginate, and chitosan.^23^

Chitosan is a natural polymeric biomaterial derived from the *N*-deacetylation process of chitin, the second most abundant polysaccharide in nature, extracted mainly from the exoskeleton of crustaceans.^24,25^ Chitosan is a polysaccharide consisting of 2-acetamido-2-deoxy-β-d-glucopyranose and 2-amino-2-deoxy-β-d-glucopyranose connected by glycosidic β linkages. It is an important biomaterial for tissue engineering due to its biocompatibility,^26^ biodegradability,^27^ broad-spectrum antimicrobial activity,^28^ analgesic effect,^29^ exudate absorption,^30^ hemostatic capacity,^31^ and low cost.^32^ Chitosan biodegradation occurs through enzymatic hydrolysis of the β glycosidic bonds connecting the *N*-acetylglucosamine and glucosamine units by lysozyme.^33^ The oligomers generated are associated with macrophage activation, promoting collagen deposition and incorporation into extracellular matrix components.^34^ Due to its cationic groups, chitosan is soluble in slightly acidic solutions^35^ and can be processed by several methods in order to produce porous membranes,^36^ films,^37^ scaffolds,^38^ or hydrogels.^39^ Several commercial chitosan-based products have been developed for wound treatment, such as Chitoflex, Celox-A,^40^ HemCon Bandage, and AX-Surgi.^41^ However, they are used in trauma contexts due to their hemostatic capacity, and modifications are desired for their use in the treatment of chronic injuries.

In order to improve the characteristics of the chitosan scaffolds (CS), *Vitis vinifera* (grape) seed oil (grape seed oil (GSO)) is incorporated into the polymeric matrix. GSO is derived from the reuse of the waste wine industry.^48^ These seeds have a content of ∼14% oil, mainly composed of unsaturated fatty acids^49^ including linoleic (67.39%) and oleic (19.06%), as well as saturated fatty acids such as palmitic acid (8.03%).^50^ GSO also contains phenolic compounds such as proanthocyanidins,^51^ phytosterols, including β-sitosterol, campesterol, and stigmasterol; and vitamin E constituents, such as tocopherols and tocotrienes, mainly α-tocopherol and γ-tocotrienol.^52^ Palmitoleic and oleic acids present in GSO are associated with anti-inflammatory processes in endothelial cells, with palmitoleic acid appearing to promote the differentiation of primary macrophages into the M2 anti-inflammatory phenotype.^53^ Linoleic acid is an essential fatty acid that plays an important chemotactic role in macrophages and induces granulation, in addition to having antimicrobial capacity against *Staphylococcus aureus*,^54^ *S. mutans*, and *S. gordonii*.^55^ Phenolic compounds such as resveratrol and proanthocyanidins have anti-inflammatory properties,^57^ a property also observed in phytosterols.^58^ Thus, GSO has antioxidant, anti-inflammatory, and antimicrobial properties, contributing to collagen production and wound regeneration,^59^ with in vivo tests demonstrating a faster rate of wound closure.^60,61^

Scaffolds developed for skin repair must present morphological characteristics that contribute to regeneration. Physical aspects, such as surface roughness, wettability, porosity, and pore interconnectivity, influence cell behavior by modulating adhesion, morphology, alignment, migration, proliferation, and gene expression.^42–44^ Control over the material’s pores depends on the manufacturing method and is important in wound healing because it allows cell growth and nutrient and fluid transport through the structure, with a suitable porosity of 60–90%.^45^ Freeze-drying is a versatile technique that allows the development of porous materials with control over porosity, pore size, pore morphology, and interconnectivity.^46^ It is based on sublimating solvents at low temperatures in a process that occurs in three stages, mainly controlled by five variables: freezing rate, freeze temperature, concentration of components, type of solvent, and components. This technique allows temperature-sensitive products not to decompose.^47^

In this article, porous scaffolds of chitosan containing different amounts of GSO were developed by freeze-drying, aiming at wound healing applications. The physical, chemical, and in vitro biological properties of the scaffolds were analyzed considering the effect of the oil content in the scaffolds. The overall results suggest that the oil tuned the morphological characteristics of the scaffolds without changes in freeze-drying parameters, being a promising biomaterial for wound healing.

## 2 Results

### 2.1 Rheological Behavior of the Hydrogels

Amplitude sweep tests were performed to determine the storage (*G*′) and loss (*G*″) moduli of the hydrogels at low and standard frequencies (0.1 and 10 rad·s^–1^) at room temperature (25 °C) within the strain range of 0.01% to 10%. Figure 1a shows the amplitude sweep test for 0.1 rad·s^–1^. All samples demonstrated a predominance of elastic over viscous behavior, which indicates the formation of a physical hydrogel. A decrease in storage modulus (*G*′) was observed with the increase in GSO, with values up to 228 Pa for CGSO6, when compared to CS (350 Pa). A reduction in the loss modulus (*G*″) for CGSO6 was also observed. The linear viscoelastic region (LVR) (LVER) extended for all compositions from 0.02% to 1%. The end of this region defines the yield point, where the hydrogel microstructure changes, corresponding to the beginning of the flow. There was no crossover point at the applied deformations. Figure 1b shows the amplitude sweep test for 10 rad·s^–1^, in which an increase in the storage and loss moduli can be observed for all samples, compared to the test at 0.1 rad·s^–1^. These results are consistent with those obtained at 0.1 rad·s^–1^, confirming there was a predominance of the elastic character for all hydrogels, a decrease in *G*′ with the addition of GSO, a decrease in *G*″ for GSO6, and an LVR between 0.06% and 1%.

**Figure 1:**
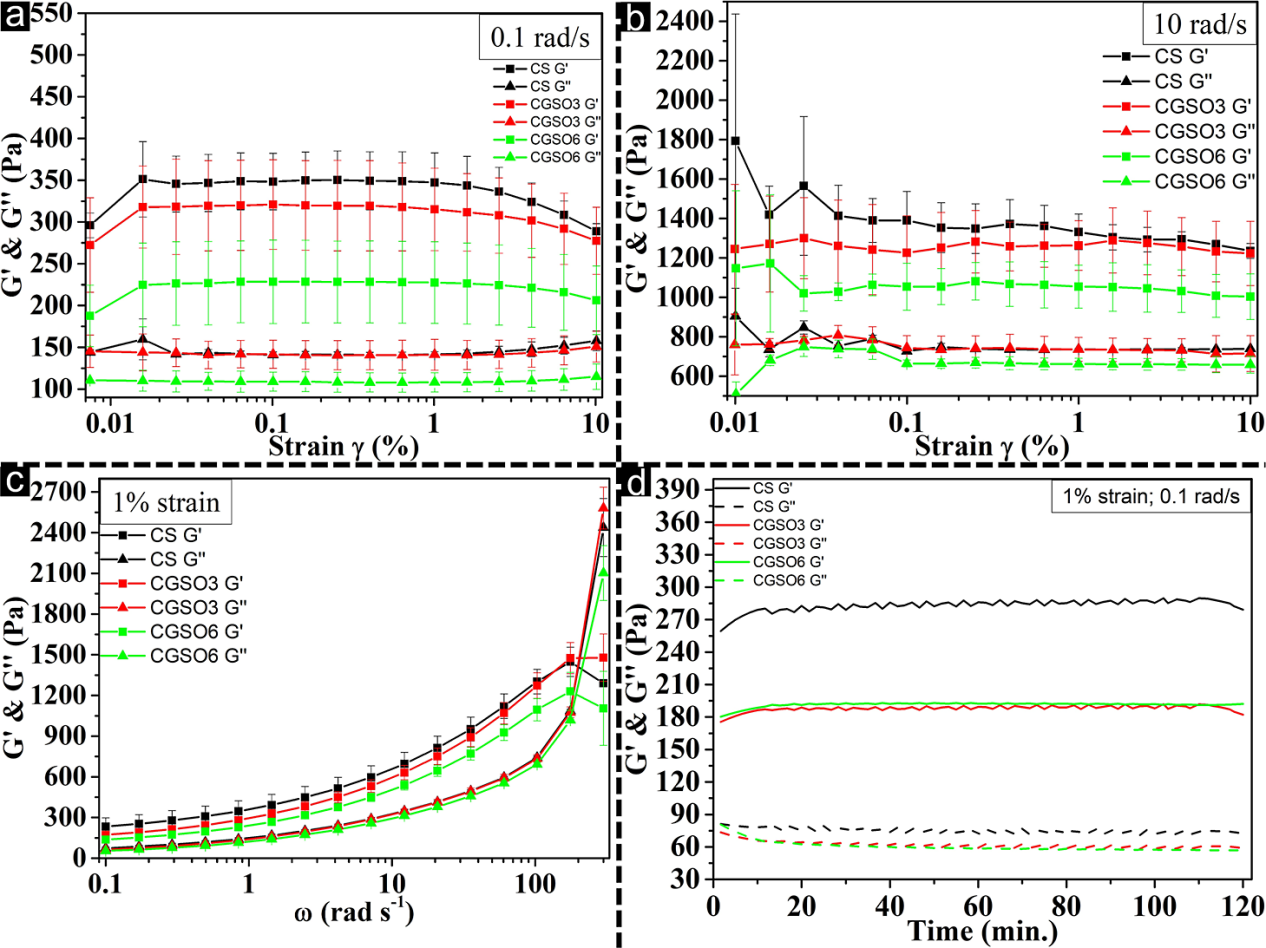
Rheological characterization of CS, CGSO3, and CGSO6 hydrogels: (a) strain sweep at 0.1 rad·s^–1^; (b) strain sweep at 10 rad·s^–1^; (c) frequency sweep at 1% strain within LVER; and (d) smoothed time sweep at 1% strain and 0.1 rad·s^–1^.

Figure 1c shows the frequency sweep plots at 1% strain. All the hydrogel compositions exhibited *G*′ values greater than *G*″ in the range from 0.1 to 200 rad·s^–1^. All samples showed an increase in both *G*′ and *G*″ with increasing frequency, a characteristic feature of viscoelastic materials. A progressive decrease in *G*′ as the GSO concentration increased was observed. All compositions exhibited a crossover point (*G*′ = *G*″) at approximately 200 rad·s^–1^, with viscous behavior becoming predominant at frequencies above this point, such as at 300 rad·s^–1^.

The time-dependent stability of the hydrogels was determined through time sweep tests (Figure 1d) at constant 1% strain and 0.1 rad·s^–1^, parameters determined from the LVR and frequency sweep tests. For all hydrogels, a predominant elastic behavior was observed throughout the 120 min test. Both *G*′ and *G*″ remained stable throughout the test for all samples. The addition of GSO (CGSO3 and CGSO6) resulted in a decrease in *G*′ and *G*″ (∼189 and ∼60 Pa, respectively), compared to the control chitosan hydrogel (∼283 and ∼72 Pa, respectively). These results demonstrate the stability of the hydrogels over time.

### 2.2 Macroscopic Aspects and Morphological Analyses of the Scaffolds

The scaffolds were produced by freeze-drying hydrogels containing 5% chitosan in an acidic medium, containing GSO at concentrations of 0.3% and 0.6% (named as CS, CGSO3, and CGSO6, respectively). After being freeze-dried, the scaffolds were submerged in an alkaline solution to thoroughly remove traces of acetic acid. The neutralization process is important to prevent acid residues from causing toxicity to cells, promote polymer chain interaction, enhancing compaction,^63,64^ and keep the biomaterial stable in an aqueous medium without the use of cross-linkers. After neutralization, the macroscopic aspects were evaluated (Figure 2, left column). The dried scaffolds presented opacity, yellowish coloration, homogeneity, and surface roughness. Moreover, they did not present an oily tactile perception, even at a higher GSO concentration.

**Figure 2:**
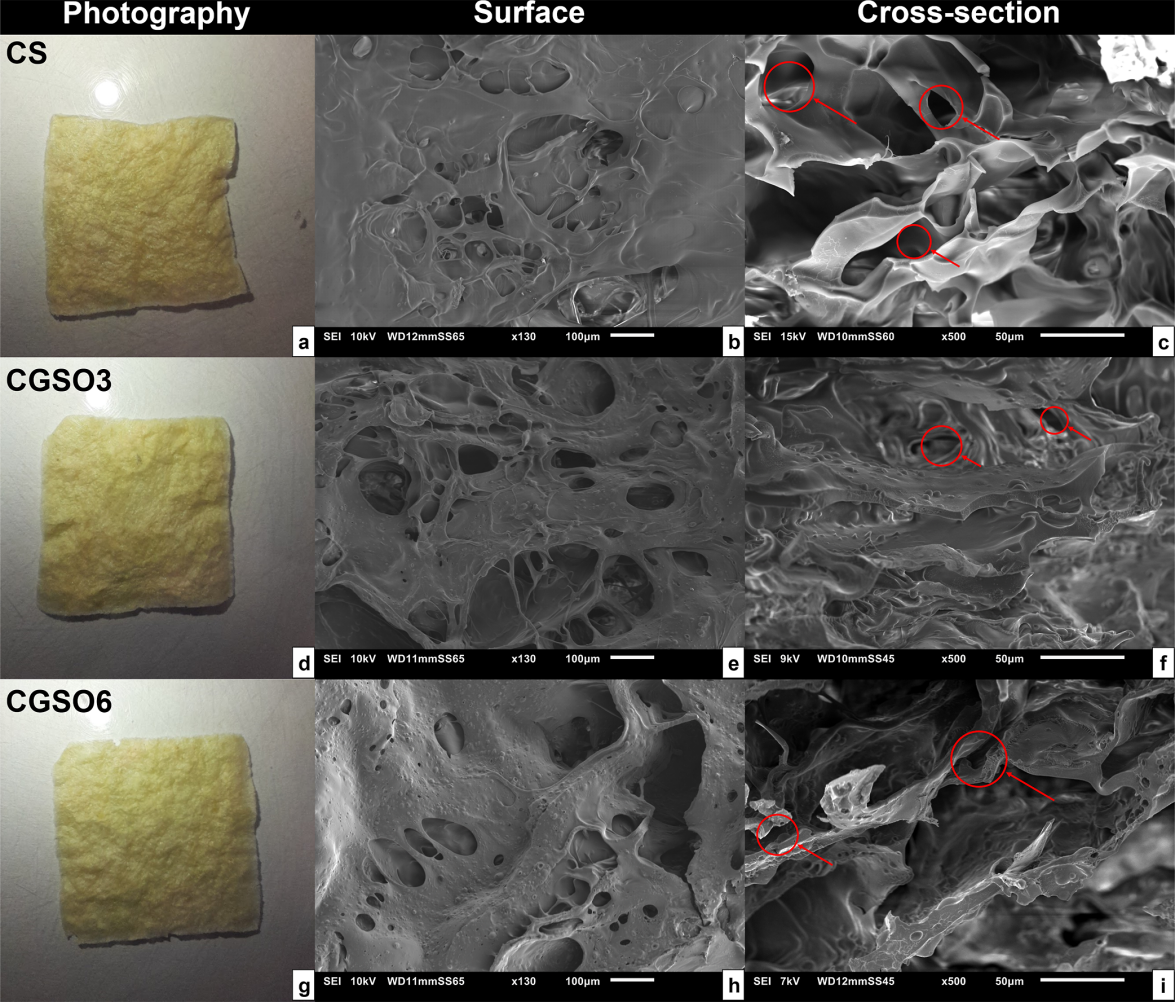
Macroscopic and scanning electron microscopy (SEM) images. Left column: macroscopic; middle column: surface morphology; right column: cross-section analysis of CS scaffolds (a–c), CGSO3 scaffolds (d–f), and CGSO6 scaffolds (g–i). Red arrows highlight the interconnection between pores.

SEM images of the scaffolds’ surface (Figure 2, middle column) indicate an irregular morphology with the presence of irregular and heterogeneous pores; the appearance of agglomerates, identified as oil droplets, in CGSO3 (not present in CS); and an increase in these structures in CGSO6. These observations indicate the incorporation of GSO adsorbed on the scaffolds’ surface and a greater surface availability of the oil with the increase in GSO used during the production of the scaffolds. Figure 2, right column, presents the SEM cross-section image of the scaffolds where the pore interconnectivity can be seen through the thickness of the scaffolds; in addition, the presence of GSO can also be observed inside the scaffolds (Figure 1f,i). The stratified structure observed in the cross-section images, common in freeze-dried materials,^116^ together with the considerably spaced interconnected pores, may provide an environment susceptible to cell growth, angiogenesis, and nutrient diffusion.^79^

### 2.3 Chemical Interactions between CS and GSO

The scaffolds were analyzed by ATR-FTIR to evaluate the incorporation of GSO within its chitosan polymeric matrix along with CS and GSO controls (Figure 3). The characteristic bands of chitosan are N–H stretch at 3360 cm^–1^ from amine groups; O–H stretch up to 3288 cm^–1^ from hydroxyl groups present in the acetyl groups of chitosan; C=O stretching at 1646 cm^–1^ and N–H bending at 1558 cm^–1^ from amide groups present in unacetylated regions of chitosan, respectively;^65,66^ C–O–C asymmetric stretch at 1150 cm^–1^ from β-(1 → 4) glycosidic linkages;^67^ and C–O stretch at 1058–1028 cm^–1^ ^68^ and C–H out-of-plane bending at 894 cm^–1^ from glycosidic linkages of monosaccharides.^69^

**Figure 3:**
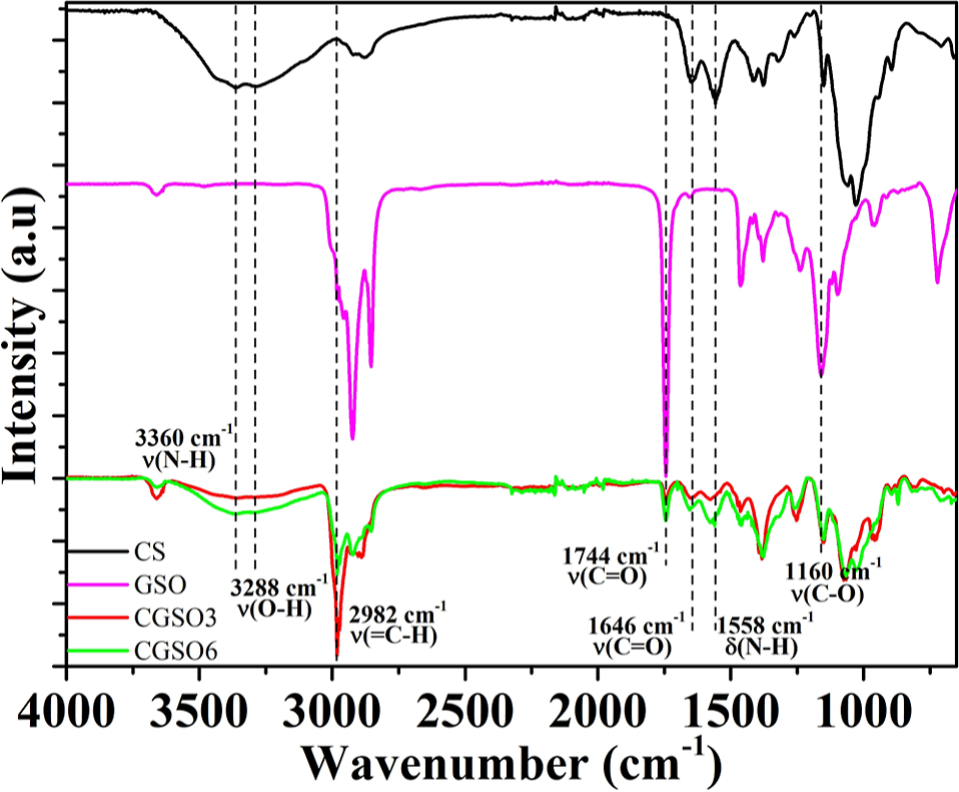
ATR-FTIR spectra of CS, GSO, CGSO3, and CGSO6, normalized at 1378 cm^–1^.

The characteristic bands of GSO are =C–H stretch at 2982 cm^–1^ from the cis configuration of unsaturated fatty acids in GSO,^70^ including palmitoleic, oleic, and linoleic acid;^71^ C–H stretch at 2924 cm^–1^ and 2854 cm^–1^ from CH_3_ and CH_2_ groups of aliphatic triglycerides, respectively;^72^ and C=O stretch at 1744 cm^–1^ and C–O deformation at 1160 cm^–1^, probably from esters such as triglycerides present in GSO.^73–75^

The IR bands observed for the CGSO3 and CGSO6 scaffolds are an overlap of the bands found for CS and GSO, which demonstrates the incorporation of the oil into the polymer matrix, as seen in the SEM images. Despite the similarities, there was the suppression of the N–H stretching bands associated with the primary amine of chitosan and the emergence of a single band in the range of 3360–3288 cm^–1^. In amines, the N–H stretching occurs in the range of 3500 to 3300 cm^–1^, with two bands in primary amines and only one in secondary amines.^76^ Therefore, the joining of the two bands into just one may indicate a strong interaction of the amine groups present in chitosan with polar groups, such as carboxylic groups, present in the oil.

### 2.4 Swelling Behavior

The swelling behavior of the scaffolds was evaluated in a phosphate buffer solution (PBS) solution at pH 7.4, simulating the conditions of the human body and aiming to analyze the fluid absorption capacity. Figure 4 shows the swelling of the CS, CGSO3, and CGSO6 scaffolds over 168 h. During the experiment, the scaffolds appeared solid with no macroscopic signs of degradation, demonstrating stability at pH 7.4.

**Figure 4:**
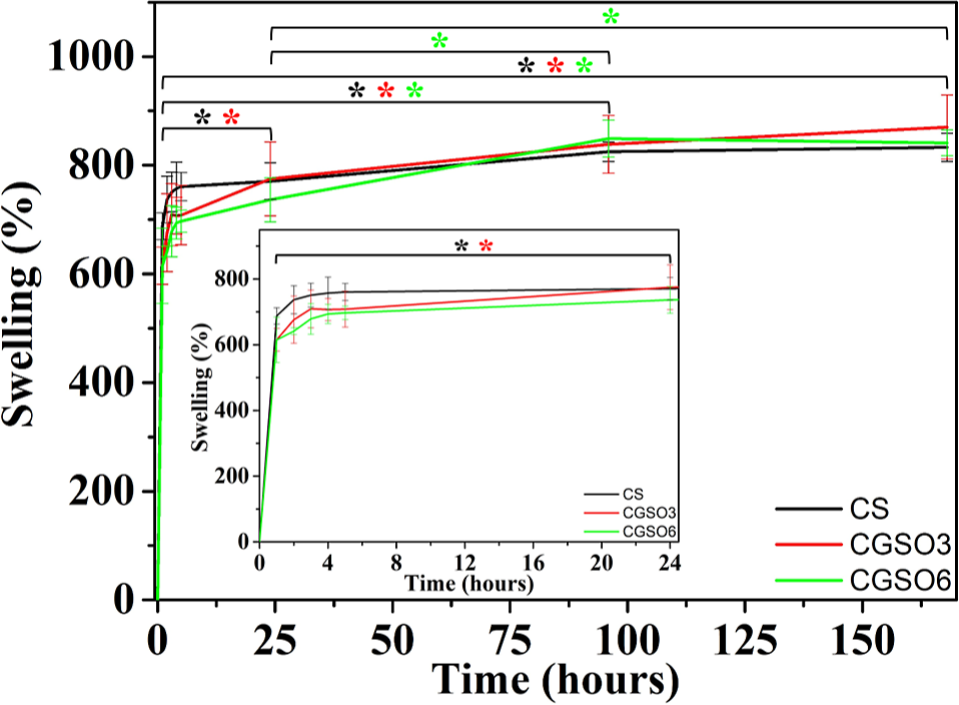
Swelling behavior of CS, CGSO3, and CGSO6 scaffolds in PBS solution up to 168 h. Inset shows the first 24 h of swelling. * Denotes statistically significant differences between samples (*p* < 0.05).

All scaffolds exhibited high swelling within the first hour, with increases greater than 600% (inset in Figure 4). In the first 96 h, the swelling continued, with a significant increase in all groups (*p* < 0.05). Between 96 and 168 h, there was a nonsignificant mean increase in swelling observed in CS (Δ = +7.8%, *q* = 0.61, *p* > 0.05) and CGSO3 (Δ = +31.7%, *q* = 0.98, *p* > 0.05), while for CGSO6, there was a nonsignificant decrease (Δ = −7.8%, *q* = 0.46, *p* > 0.05), causing all scaffolds to exceed 800% swelling and indicating a maximum swelling level after 96 h.

Within the first 5 h, the scaffolds containing GSO presented numerically lower swelling averages than those seen in CS. Although they were not statistically different, these values suggest a trend of lower water absorption, consistent with the hydrophobic nature of the GSO adsorbed on the surface of the biomaterial, as seen by SEM. This lower water absorption by chitosan materials containing oil is portrayed in the literature.^77,78^ At times longer than 24 h, the swelling observed for all scaffolds is not significantly different between them, indicating that despite the different kinetics, they reach the same swelling values.

### 2.5 Scaffolds’ Porosity

Figure 5 presents the porosity of the scaffolds determined by the liquid displacement method. The pores facilitate the integration between the biomaterial and cells, promote the dispersion of nutrients and oxygen throughout the tissue, and favor angiogenesis.^79^ Therefore, porosity is an important parameter for promoting cell growth.^80^ The porosity of the scaffolds increased with higher GSO content: the estimated percentage values were 53 ± 6, 60 ± 4, and 70 ± 7 (CS, CGSO3, and CGSO6, respectively), with a significant increase (*q* = 4.31, *p* < 0.05) between CS and CGSO6.

**Figure 5:**
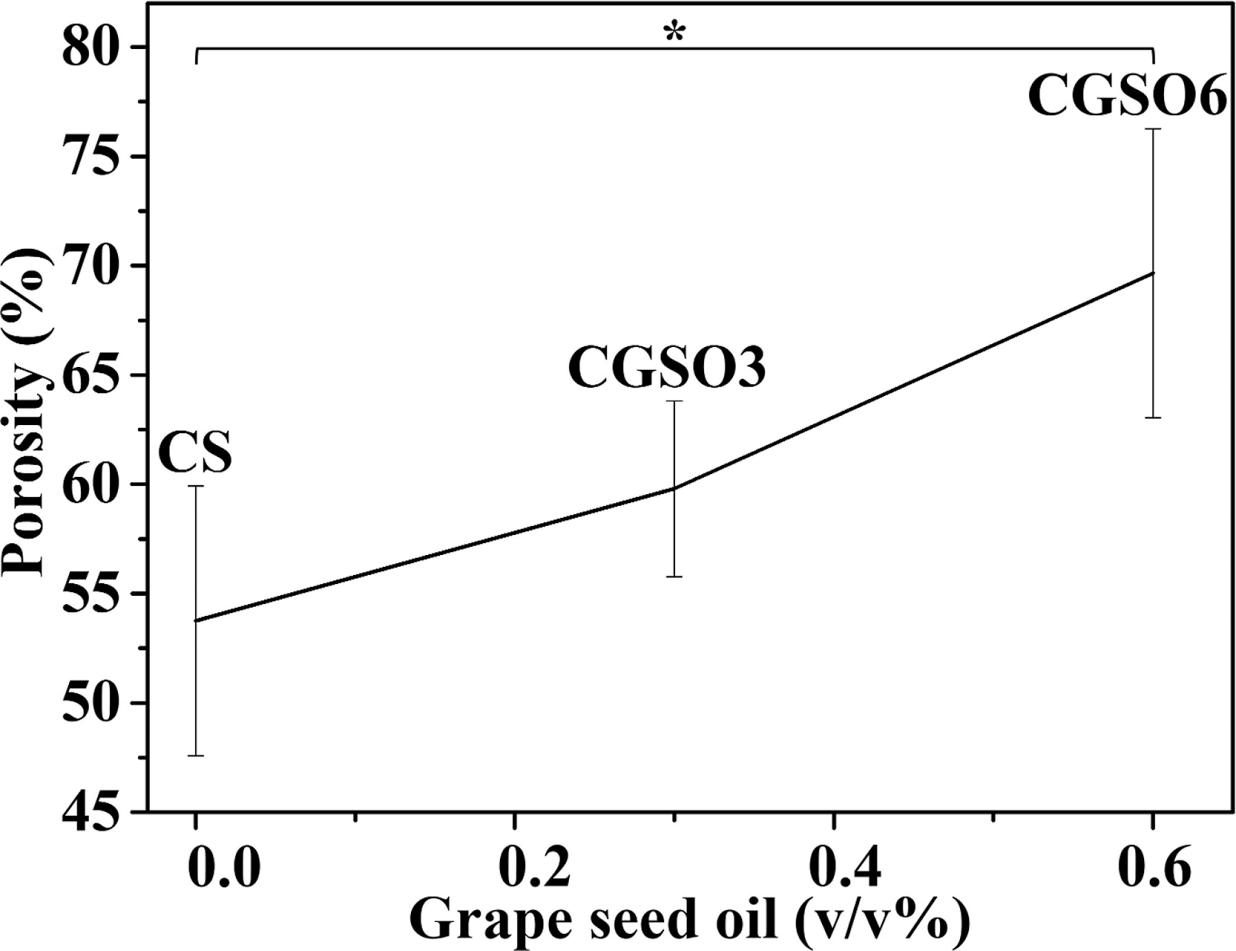
Effect of GSO concentration on the porosity of the scaffolds. * Denotes statistically significant differences (*p* < 0.05).

In addition to the liquid displacement assay, micro-CT imaging was performed to assess the internal microstructure of the scaffolds, as shown in Figure 6. Based on the reconstructed cross-sectional images (Figure 6, left column), the porosity percentages were estimated as 59%, 62%, and 67% for CS, CGSO3, and CGSO6 scaffolds, respectively, in agreement with the values obtained from the liquid displacement method. All scaffolds exhibited an interconnected pore network, as observed in the magnified sections. The pores did not display a regular geometry, making the comparison of the pore area more relevant than direct size measurements. Nevertheless, scaffolds containing GSO presented regions with a more organized lamellar pore arrangement in contrast to the predominantly random pore structure observed in the CS scaffold.

**Figure 6:**
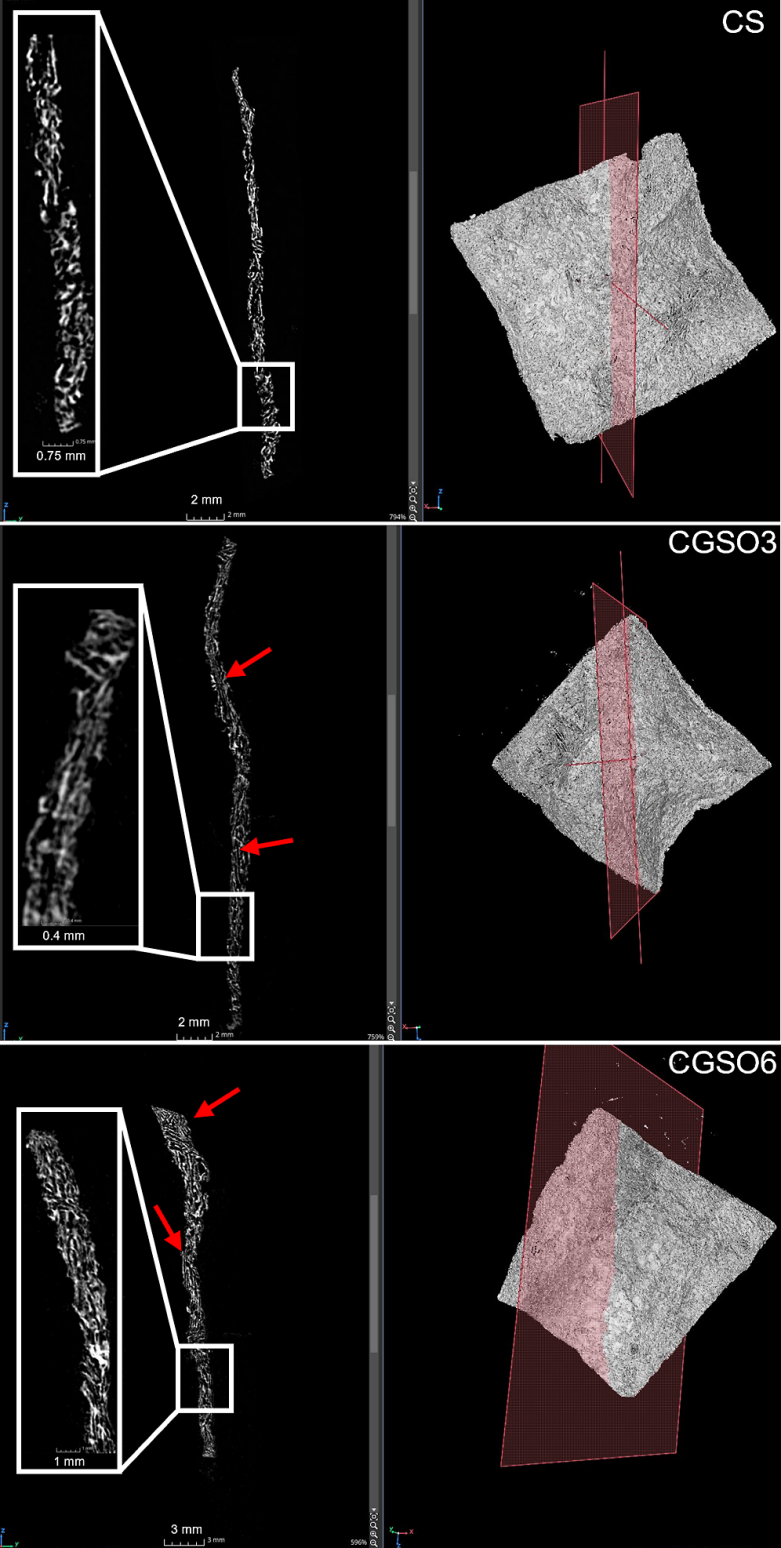
Micro-CT reconstructions of CS, CGSO3, and CGSO6 scaffolds. Right: 3D volume renderings indicating the position of the selected cross-section (red plane). Left: cross-sectional images with magnified regions highlighting the internal pore morphology. Red arrows: lamellar pore structures.

### 2.6 In Vitro Enzymatic Degradation of the Scaffolds

Enzymatic degradation experiments were performed in PBS solution at pH 7.4 containing 13 mg/L of lysozyme, which is the highest concentration of the enzyme found in the human body,^81^ at a temperature of 37 °C at intervals of 3, 7, 21, and 28 days. Lysozyme is responsible for the catalytic hydrolysis of the β(1 → 4) bonds between the *N*-acetylglucosamine and glucosamine groups; the hydrolysis is inversely proportional to the degree of deacetylation, molecular weight (MW), and crystallinity.^33^ Due to the hydrophobic nature of GSO dispersed in the scaffolds, the degradation process of the CGSO3 and CGSO6 scaffolds is expected to be slower when compared to that observed for CS, due to the smaller surface area available for direct interaction with the enzyme.

Figure 7 shows degradation of the scaffolds over time. It can be seen that there is a significant mass loss (*p* < 0.05) for the scaffolds up to 21 days, with values remaining statistically equivalent between 21 and 28 days. The scaffolds presented maximum degradation values of 12.4% ± 0.6%, 12.4% ± 0.5%, and 11% ± 2% for the CS, CGSO3, and CGSO6 scaffolds, respectively. A tendency to lower degradation values can be observed with the addition of GSO into the scaffolds, as it is noticeable in CGSO6 compared to CS. This behavior is in accordance with the hydrophobic nature of GSO present in the scaffolds and has been reported in the literature, where lower mass loss is observed for biomaterials containing natural oils when compared to those composed of a pure polymeric matrix.^82,83^

**Figure 7:**
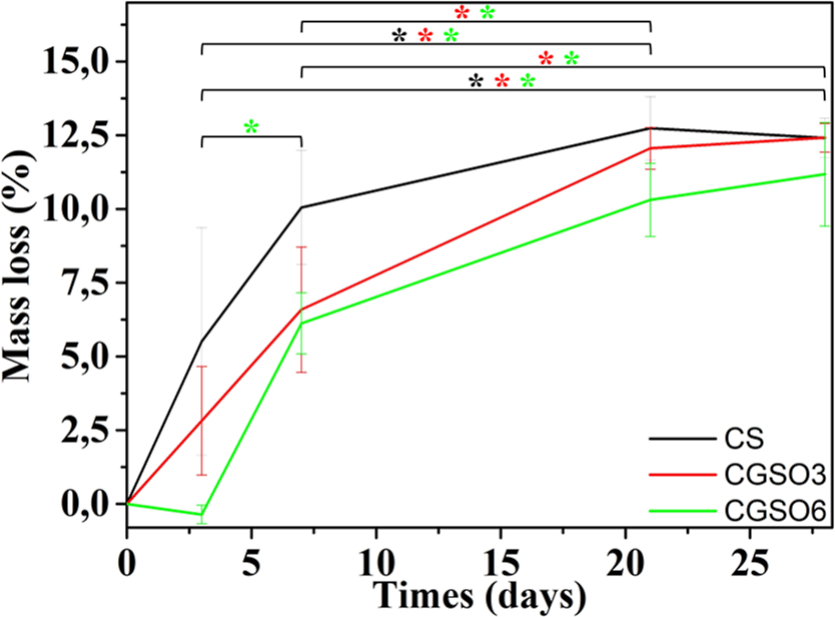
In vitro enzymatic degradation of CS, CGSO3, and CGSO6 in PBS + lysozyme solution over 28 days. * Denotes statistically significant differences (*p* < 0.05).

### 2.7 Cytotoxicity of GSO

Vero cells were exposed for 24 h to five different concentrations of GSO (0.032, 0.075, 0.15, 0.3, and 0.6%; v/v), preconditioned for the same period. Figure 8a shows cell integrity, determined by the crystal violet method, after 24 h of contact with the GSO solutions. Although the median values indicate lower cell integrity compared to the conditioned medium control (CMC), the interquartile range box demonstrates no statistically significant variation, indicating that GSO does not have a cytotoxic effect on Vero cells. Such behavior is reported in the literature for other natural oils.^84,85^ Figure 8b shows the absorbance after the conversion of resazurin to resorufin by NADH dehydrogenases, associated with the mitochondrial metabolism of the cells.^86^ There is an increase in cellular metabolism in the presence of GSO, with metabolic activities slightly higher than those of CMC (0.032, 0.075, and 0.15%; v/v) and significantly higher at concentrations of 0.3% and 0.6% (v/v) of GSO.

**Figure 8:**
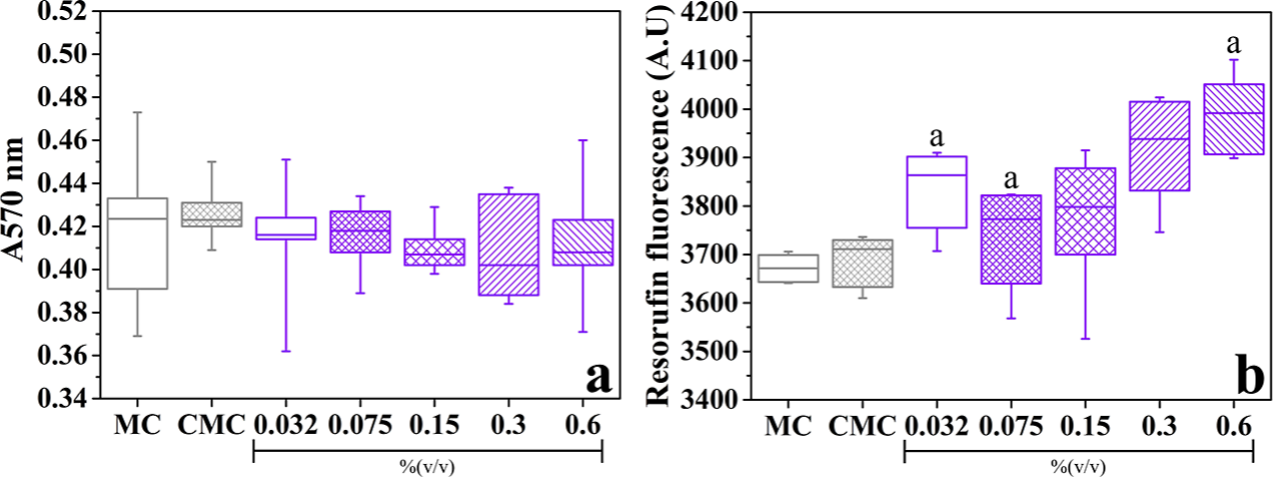
Indirect cytotoxicity of GSO at different concentrations. Vero cell integrity (a) and cell metabolism (b), assessed by crystal violet and resazurin assays, respectively, a: significant difference in relation to CMC.

### 2.8 Indirect Cytotoxicity of the Scaffolds

The scaffolds were conditioned for 24 h, and then the scaffold-conditioned medium was used in Vero cell culture for 24 h. Figure 9a shows the cellular integrity, determined by a crystal violet assay. The results reveal a small reduction in cellular integrity for cells in contact with the solutions incubated with the CS, CGSO3, and CGSO6 compared to the CMC. There were no significant differences in cellular integrity between the scaffolds, demonstrating that GSO does not directly affect cellular integrity and does not present a negative synergistic effect with chitosan.

**Figure 9:**
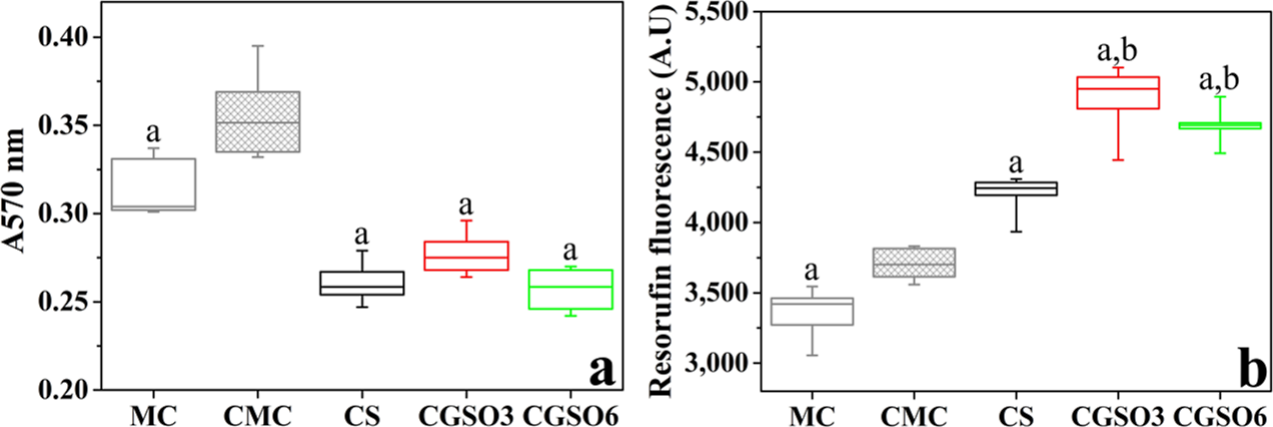
Indirect cytotoxicity of the scaffolds at different concentrations. Vero cell integrity (a) and cell metabolism (b), assessed by crystal violet and resazurin assays, respectively. Significant differences: a: in relation to CMC; b: in relation to CS.

Figure 9b shows the results of the resazurin assay for the scaffolds. An increase in cellular metabolism was observed for scaffolds when compared to the CMC (*p* < 0.05). The scaffolds containing GSO (CGSO3 and CGSO6) exhibited significantly higher metabolic activity than CS (*p* < 0.05) and were statistically similar to each other.

## 3 Discussion

Skin acts as the primary barrier of the human body and is susceptible to various external and internal damages caused by mechanical trauma, infections, or inflammations that, when they disrupt the normal anatomical structure, result in lesions.^6,87^ Despite the innate regenerative capacity of the skin, deep wounds or chronic inflammations such as bedsores, burns, and diabetic ulcers have limited regenerative capacity.^15^ Novel biomaterials are required to promote regeneration of these lesions. These materials must act as a barrier against lesions and microorganisms, have anti-inflammatory properties, tailored degradation, and have adequate porosity, enabling the exchange of gases, nutrients, metabolites, and cell migration.^20–22,88^ GSO is a compound-rich in unsaturated fatty acids^49^ such as oleic and palmitoleic acids, associated with anti-inflammatory processes;^53^ linoleic acid, capable of inducing the granulation stage;^54^ and antioxidants that act by reducing oxidative stress.^57^ Although chitosan has been clinically used with suitable results, its properties can be tuned by GSO incorporation into the polymeric matrix. Thus, the chitosan-based porous scaffolds with GSO developed in this work aim to mimic the extracellular environment, providing a controlled humidity, along with the potential bioactive benefits of GSO.

The stability of hydrogels produced without the use of chemical cross-linkers was evaluated by rheological tests of amplitude, frequency, and time sweeps. Amplitude sweep tests (Figure 1a,b) revealed that all compositions exhibited predominantly elastic behavior (*G*′ > *G*″),^115^ which is characteristic of physical hydrogels.^106^ However, an increase in the GSO concentration decreased the storage modulus (*G*′), suggesting a possible plasticizing effect of the oil,^107^ thereby reducing the interactions between the chitosan polymer chains and increasing chain mobility.^108^ A decrease in the loss modulus (*G*″) was also observed, possibly due to the lubricating effect of the oil, which reduces internal friction between the polymer chains.^109^ At higher frequencies (10 rad·s^–1^), both *G*′ and *G*″ increased, as expected for physical hydrogels.^110^ Frequency sweep tests (performed within the LVER at 1% strain) (Figure 1c) confirmed the dominance of elastic behavior and the inverse relationship between GSO concentration and *G*′, corroborating that GSO reduces hydrogel stiffness.^111^ The increase in *G*′ with frequency characterizes them as physical hydrogels.^110^ Furthermore, the crossover point (*G*′ = *G*″) occurred only at higher frequencies, demonstrating the stability of the gels at low frequencies. Finally, the time sweep test (Figure 1d) demonstrated the stability of the hydrogels over 120 min.

The scaffolds (CS, CGSO3, and CGSO6) were manufactured by freeze-drying, aiming materials with high porosity and variable pore sizes. The freeze-drying process allows the fabrication of porous scaffolds from hydrogels, where the ice crystals in the hydrogel sublimate without the collapse of the microstructure. Among the main processing parameters that affect the morphology of the scaffolds are the freezing temperature, freezing rate, polymer concentration, and viscosity of the solution.^47^ Our scaffolds were developed from reagents with functional properties, aiming at tunable properties, given by the concentration of GSO, and without the need for additional freezing methods, which would increase the manufacturing complexity. The scaffolds presented an apparent rough and homogeneous surface without an oily feel to the touch. The ATR-FTIR data demonstrate that the GSO was effectively incorporated into the polymer matrix (Figure 3), as shown by the appearance of the characteristic bands of pure GSO in CGSO3 and CGSO6 scaffolds and by the coalescence of the N–H stretching bands into a single broad absorption in the range of 3360–3288 cm^–1^. These spectral changes suggest the formation of strong interactions that may stabilize the chitosan–GSO network of the scaffolds. Surface SEM analysis shows pores oriented toward the interior of the scaffold and confirms the presence of GSO. Cross-sectional SEM images show that the pores are distributed throughout the structure in an interconnected network and that GSO is incorporated homogeneously throughout the sample, observed as distinct oil droplets (Figure 2). The micro-CT images (Figure 6) reveal the pore interconnectivity and show that the addition of GSO favored the formation of the lamellar pore structure on the scaffolds. Pore interconnectivity is an essential parameter for scaffolds, improving permeability, facilitating the transport of fluids, nutrients, and biomolecules and the removal of debris through the structure thickness.^89^

Overall porosity measure showed that CGSO3 and CGSO6 presented porosity mean values of 60% and 70%, respectively, higher than that observed for CS scaffolds (53%), with a significant increase between CS and CGSO6 (Figure 5). Biomaterials with porosity values between 60% and 90% promote the healing process,^45^ recent studies demonstrate that freeze-drying generates highly porous materials for wound healing, including those based on chitosan and carboxymethyl cellulose (64–71% porosity),^112^ hyaluronic acid and hydroxypropyl chitosan (70–78% porosity),^113^ and κ-carrageenan (85–95% porosity).^114^ In line with these studies, the incorporation of GSO into the chitosan matrix can be used to provide physically tailored morphological characteristics to the scaffolds that are suitable for skin repair without requiring processing changes. The increase in porosity of the oil-containing scaffolds can be explained by the coalescence of oil droplets during the water-freezing process, as seen in water-in-oil-in-water (W/O/W) emulsions,^100^ resulting in greater porosity with the increasing of GSO concentration into the chitosan matrix. The literature indicates that porosity is one of the main factors that determine biocompatibility of biomaterials, and its increase is associated with a series of pro-healing effects such as the reduction of foreign body reactions and modulation of effects such as angiogenesis, cell migration, oxygen flow, and macrophage polarization, in which an increase in porosity can promote the differentiation of the anti-inflammatory M2 phenotype.^79^

Permeability is influenced by porosity, interconnectivity of materials, and hydrophilicity of biomaterials, enhancing the effect of capillarity on exudate absorption.^90,91^ The three developed scaffolds (CS, CGSO3, and CGSO6) showed great fluid absorption capacity due to the hydrophilic nature of chitosan (Figure 4). Swelling reached up to 800% in a period of 7 days, a value comparable to those of other porous polysaccharides. Wound healing biomaterials such as chitosan and carboxymethyl cellulose,^112^ hyaluronic acid and hydroxypropyl chitosan,^113^ and κ-carrageenan^114^ exhibit absorptions of 700–2000%,^112^ 2200–3200%,^113^ and 700–3000%,^114^ respectively. This swelling is responsible for maintaining the shape of the scaffolds over time and highlighting the importance of the neutralization step in non-cross-linked CS. This notable shape retention is a direct consequence of the scaffold neutralization process in a NaOH solution. This treatment deprotonates residual ammonium groups (NH_3_^+^) to their corresponding primary amines (NH_2_), yielding a more rigid and compact structure. The resulting deprotonated amines render the chitosan insoluble at pH 7.4, which possibly reduces its hydrophilicity and obviates the need for additional cross-linkers.^35,105^ The swelling during the first 5 h for the CGSO3 and CGSO6 scaffolds presented numerical averages lower than those of CS; however, the values were statistically equivalent between them throughout the 7 days of testing. The similar swelling values observed in the scaffolds after 7 days, leading to similar fluid absorption behavior, may be due to the antagonistic effect of two factors. The first is the hydrophobic nature of GSO, as evidenced by the slower absorption kinetics for scaffolds containing GSO in the apparent contact angle test (Figure S1) and by its dispersion on the surface of the scaffolds (CGSO3 and CGSO6), limiting the fluid absorption capacity, as observed in the SEM images. The second is the increased porosity observed in CGSO3 and CGSO6, which favors swelling. During the regeneration process of skin lesions, it is important to control exudate, an early inflammatory reaction. In chronic wounds, excessive exudate can increase the risk of infection, prolong the inflammatory phase, inhibit the formation of blood vessels and re-epithelialization, and cause edema in granulation tissues.^92^ Therefore, the swelling behavior observed in the developed scaffolds suggests a suitable capacity for the absorption of excess exudate, which may contribute to preventing the prolongation of the inflammatory process, maintaining moisture in the injured area, and potentially supporting skin tissue repair.

No significant differences were observed in the loss of mass between the scaffolds over 28 days, with a maximum average degradation of ∼12% in 28 days (Figure 7). At the end of the experiments, the scaffolds did not appear gelled, retaining their structural integrity despite the degradation, possibly due to the chitosan neutralization step. Considering specific times, lower average values of degradation were observed for the scaffolds with GSO, especially CGSO6, when compared to CS. As evidenced by slower water absorption kinetics for CGSO3 and CGSO6 compared to CS (Figure S1), this behavior may have been caused by the hydrophobic oil droplets dispersed on the external and internal parts of the scaffold, as observed by the SEM surface and cross-section images. The presence of the oil possibly inhibited the direct enzymatic action of lysozyme in regions of the scaffold, reducing degradation. On the other hand, the greater porosity of the CGSO3 and CGSO6 scaffolds enables a swelling equivalent to CS, leading to a higher number of β(1 → 4) bonds available for catalytic hydrolysis between the free *N*-acetylglucosamine and glucosamine groups. These two effects may be responsible for the fact that, despite the lower average mass loss values in CGSO6 and CGSO3 compared to CS, they are not statistically different from each other.

Scaffolds for skin repair need to have a tunable degradation rate that allows mass loss synchronized with tissue regeneration.^93^ Insights into appropriate degradation rates for biomaterials can be provided by clinical trials on chronic wounds. In clinical practice, patients arrive at ambulatory clinics with chronic lesions, such as ulcers, with different persistence times, from recent cases (<6 weeks) to old ones (>24 months),^94^ and the conventional treatment of leg ulcers may not result in 100% epithelialization in a period of up to 24 weeks.^95^ Statutory health insurance data analysis indicates that almost 20% of leg ulcers or diabetic foot ulcers healed after 6 months, and the average time to closure of leg ulcers was 8.9 months.^96,97^ Commercial scaffolds made of collagen and regenerated oxidized cellulose, showed complete wound closure in 45% of patients with diabetic foot ulcers of up to 6 months duration in a 12 week period, compared with 33% of the control group (moistened gauze).^98^ A type I collagen matrix scaffold incorporated with antimicrobial polyhexamethylene biguanide (PCMP) was used in the treatment of pressure ulcers, surgical wounds, venous ulcers, and diabetic ulcers over a 12 week study period. The commercially available scaffold showed complete wound closure in 37% of cases with a mean time of 6.7 weeks.^99^ Thus, chronic wounds take a long time for complete wound closure, which can take up to 2 years due to the remodeling process,^17,18^ indicating that the degradation values obtained for the CGSO3 and CGSO6 scaffolds are consistent with other biomaterials designed to provide prolonged support.

The biocompatibility of GSO was evaluated by cellular integrity and metabolism assay at different concentrations of GSO (0.032, 0.075, 0.15, 0.3, and 0.6%; m/v; Figure 8). There was no significant difference in the cellular integrity analyzed for the different concentrations of GSO or the controls. This result agrees with the protective effect observed for thorn GSOs in pancreatic β-cells, in which there was a significant reduction in apoptosis in cells exposed to high glucose.^101^ There was a significant increase in the metabolic activity of Vero cells in contact with concentrations of 0.032, 0.3%, and 0.6% GSO. These results demonstrate that GSO does not have a significant cytotoxic effect, even at high concentrations (0.6%). However, the increase in metabolism may be associated with an increase in mitochondrial activity due to cellular stress, given that its reduction occurs by oxidoreductases present in the mitochondria.^102^ It is noted that the concentrations with the highest median (0.3% and 0.6% v/v) must be higher than the oil values released by the CGSO3 and CGSO6 scaffolds, given that after the neutralization process, there is still GSO present in the scaffolds, as seen by the FTIR results.

All scaffolds showed cellular integrity greater than 70% compared to the CMC, with no difference in cellular integrity among the three scaffolds (CS, CGSO3, and CGSO6). Cell viability values above 70% are recognized as the cytotoxicity limit,^103^ indicating that the scaffolds are not cytotoxic. The CS scaffold showed higher metabolic activity than the controls, while CGSO3 and CGSO6 had higher values than the other groups (Figure 9). This result demonstrates that the CS-conditioned medium induces cellular stress in Vero cells. The presence of GSO in CGSO3 and CGSO6 scaffolds triggers a more pronounced metabolic response despite the chitosan matrix.

## 4 Conclusion

This study evaluated the physical, chemical, and in vitro biological properties of adding GSO (0.3 and 0.6% v/v) to a chitosan matrix for the production of freeze-dried scaffolds aimed at skin repair. The materials developed enabled the tuning of the scaffolds’ properties without employing very low-temperature conditions, one of the characteristics responsible for the high energy consumption during large-scale freeze-drying processes, simply through the addition of GSO, a natural product derived from agricultural waste. This effect is likely due to the coalescence of oil droplets during the water-freezing process, optimizing the microstructure of the CGSO3 and CGSO6 scaffolds. Thus, a 17% increase in porosity was possible with the addition of 0.6% GSO. The scaffolds exhibited a stratified, interconnected porous morphology, a property known to facilitate cell growth and nutrient transport, in addition to maintaining high swelling, a degradation rate comparable to that observed in chronic wound regeneration, and nontoxicity. Moreover, the natural materials used, without any chemical cross-linker, offer a promising biomaterial for wound healing applications. Future studies are now required to investigate whether the antimicrobial activity and hemostatic capability of chitosan, along with the antioxidant, anti-inflammatory, and pro-healing properties of the GSO, can provide an accelerated healing process in chronic wounds. Further in vitro and in vivo studies, such as scratch assay, wound healing marker analysis, and the full-thickness wound model, are necessary to confirm the biological potential of these scaffolds in wound treatment. Altogether, the results demonstrate that the scaffolds of chitosan containing GSO exhibit desired properties for the fabrication of accessible scaffolds for skin repair.

## 5 Materials and Methods

### 5.1 Materials

Medium MW chitosan (Sigma-Aldrich, 448877-250G) (deacetylation ≥75%, *M*_W_ = 190–310 kDa), glacial acetic acid (Sigma-Aldrich, 695092-2.5L), GSO (Distriol, Brazil), and sodium hydroxide (NaOH) (Synth, Brazil) were used in the development of the scaffolds. To produce the phosphate buffer saline (1× PBS, pH 7.4) solution, sodium chloride (137 mM, Casa Americana, Brazil), potassium chloride (2.7 mM, Casa Americana, Brazil), disodium hydrogen phosphate (10.1 mM, ALLKIMIA, Brazil), potassium phosphate monobasic (1.8 mM, Sigma-Aldrich, P5655-500G), and hydrochloric acid (Dinâmica, Brazil) were used for pH adjustment. Absolute ethanol (Sigma-Aldrich, 459844-1L) was used for the porosity assay, and human lysozyme (Sigma-Aldrich, L1667-1G) for the in vitro enzymatic degradation assay. For biological assays, the Vero cell line (Adolfo Lutz Institute, São Paulo, Brazil), Ham-F-10 medium (Sigma-Aldrich, St. Louis, MO, USA), fetal bovine serum (FBS) (Gibco, Waltham, USA), penicillin, streptomycin, trypsin, EDTA, amphotericin B solutions (Sigma-Aldrich, St. Louis, MO, USA), and resazurin dye (7-hydroxy-3*H*-phenoxazin-3-one 10-oxide; Sigma-Aldrich, St. Louis, MO, USA) were used. All of the reagents and solvents were used as received from commercial sources without further purification.

### 5.2 Methods

#### 5.2.1 Scaffold Preparation

For CS, 5% chitosan (w/v) was dissolved in a 10% (v/v) acetic acid solution. For GSO scaffolds, 0.3% or 0.6% (v/v) GSO was added to a 10% (v/v) acetic acid solution before the addition of CS. All solutions were manually stirred until reaching complete homogeneity and stored in the dark for the next 24 h. Solutions were sonicated in a sonication bath (Q3.0/40, Ecosonics, Brazil) for 2 h, transferred into syringes, and centrifuged (Excelsa II 206-BL, Fanem, Brazil) at 3600 rpm for 30 min (2260 G), poured into 2 mm thick polypropylene molds, and frozen at −20 °C overnight. The frozen solutions were freeze-dried (FDB-5503, Operon, Korea) at −55 °C for 24 h. The dry scaffolds were neutralized in a 1 M NaOH solution for 30 min and dried at room temperature. The dry scaffolds were named CS (pure chitosan), CGSO3 (0.3% GSO), and CGSO6 (0.6% GSO).

#### 5.2.2 Rheological Properties of Hydrogels

The hydrogel rheological measurements were performed on a rheometer (Anton Paar, MCR502, Austria) using parallel-plate geometry with a 0.8 mm gap at 25 °C. A 50 mm diameter bottom plate and a 25 mm long top plate were used. Storage moduli (*G*′), loss moduli (*G*″), and LVR were found throughout amplitude sweep tests performed at a constant angular frequency of 10 rad·s^–1^ and 0.1 rad·s^1^, both over a strain range of 0.01% to 10%. Based on the LVR profile, a strain of 1% was selected for the following tests. The frequency sweep test was conducted at a constant strain of 1% within a frequency of 0.1 to 300 rad s^–1^. The temporal evolution of the viscoelastic properties was monitored via a time sweep test for 120 min at ambient temperature (25 °C), which is the typical preparation and handling conditions, with 1% of strain obtained from LVR and with 0.1 rad·s^–1^ obtained from the frequency sweep test. The amplitude and frequency sweep tests were performed in triplicate. To improve clarity, time sweep data was smoothed using OriginPro software.

#### 5.2.3 Physicochemical Characterization of Scaffolds

##### 5.2.3.1 Scanning Electron Microscopy

The morphology (surface and cross-section) of the scaffolds of different compositions was evaluated by SEM (JSM-6010LA, JEOL, Japan) using a secondary electron detector. Scaffold samples were sputter-coated (ACE200, Leica, Germany) with a 7 nm layer of gold prior to the analysis. Microscopy parameters, such as working distance and accelerating voltage, were adjusted to maximize the resolution at each magnification.

##### 5.2.3.2 Fourier Transformed Infrared Spectroscopy

The scaffolds’ functional groups were assessed by Fourier transformed infrared (FTIR) spectroscopy (Spectrum Two FT-IR, PerkinElmer, USA) using the attenuated total reflectance mode. The spectra were recorded within the range of 500–4000 cm^–1^ with a resolution of 2 cm^–1^ and 64 scans. The spectra were normalized to 1378 cm^–1^, corresponding to the symmetric deformation of CH_3_,^65^ aiming to minimize the effects of slight variations in the scaffold thickness.

##### 5.2.3.3 Swelling Behavior

The water absorption was evaluated in 1× PBS. The scaffolds were dried in an oven at 40 °C until a constant mass was achieved. The dried scaffolds were weighed (*W*_0_) and immersed in 1× PBS (7.4 pH) using a fixed sample-to-solution ratio of 1:350 (w/v), in closed containers, and kept at room temperature for up to 7 days. After 1, 2, 3, 4, 5, 6, 24, 96, and 168 h of immersion, the surface water was gently removed with absorbent paper, and the scaffolds were weighted (*W*_1_). Following the measurement, the scaffolds were returned to the same immersion medium to continue with the swelling study. The swelling ratio was calculated as presented in eq 1

1 $$SR=\frac{W_{1}-W_{0}}{W_{0}}\times 100\%$$

where *W*_0_ and *W*_1_ are the weights of dry and swollen scaffolds. Each data point was measured in triplicate.

##### 5.2.3.4 Porosity

The scaffolds’ porosity was estimated by a modified liquid displacement method.^62^ The scaffolds were cut into pieces of approximately (8 mm × 8 mm × 1 mm) with the aid of a cutting template, and their exact dimensions (*l*, *w*, and *t*) were measured using a caliper. The pieces were then dried in an oven at 40 °C until a constant mass was achieved. The dry scaffolds were weighed (*W*_d_) and immersed in absolute ethanol using a fixed sample-to-solution ratio of 1:200 (w/v) in closed containers and kept at room temperature for 24 h, assuming a density of 0.789 g/cm^3^. After this period, the surface ethanol was gently removed using absorbent paper, and the scaffolds were weighted (*W*_w_). The porosity (%) is calculated using eq 2.

2 $$Porosity\;\left(\%\right)=\frac{W_{w}-W_{d}}{\rho \left(l\times w\times t\right)}$$

where *W*_w_ and *W*_d_ are the dry and wet weights of the scaffolds, respectively; ρ is the density of absolute ethanol; and the length, width, and thickness are represented by *l*, *w*, and *t*, respectively. Each data point was taken in triplicate.

The internal microstructure of the scaffolds was analyzed using a microcomputed tomography system (Metrotom 800, Zeiss, Germany). Samples were scanned at a voltage of 100 kV and a current of 160 μA, with a voxel size of 26 μm, with 1200 projections, 267 ms integration time, and 8× gain. For quantitative analysis, the reconstructed slices were processed using VGStudio Max 2022.1 and ImageJ softwares.

##### 5.2.3.5 In Vitro Enzymatic Degradation

The dry scaffolds were weighed (*W*_0_), immersed in 35 mL of 1× PBS solution (7.4 pH) containing 13 mg/L lysozyme, and incubated at 37 °C for different periods (3, 7, 21, and 28 days). Twice a week, the medium pH was evaluated and replaced with a fresh solution. At the given periods, the scaffolds were removed, washed with deionized water, and frozen at −20 °C. Thereafter, the frozen scaffolds were freeze-dried, and the final weight (*W*_1_) was evaluated. The degradation (%) is calculated using eq 3.

3 $$Degradation\;\left(\%\right)=\frac{W_{0}-W_{1}}{W_{0}}\times 100\%$$

where *W*_0_ and *W*_1_ are the weights of dry scaffold before and after degradation, respectively.

#### 5.2.4 Biological Characterization

##### 5.2.4.1 Cell Line, Culture, and Maintenance

The Vero cell line, from kidney cells of the African green monkey (*Cercopithecus aethiops*, CCIAL 057, provided by the Adolfo Lutz Institute, São Paulo, Brazil), was used in the present study. Cells were maintained in Ham-F-10 medium (Sigma-Aldrich, St. Louis, MO, USA), supplemented with 10% FBS (Gibco, Waltham, USA), 1% (v/v) of 10000 U mL^–1^ penicillin, 10 mg.mL^–1^ streptomycin, and 25 μg.mL^–1^ amphotericin B solutions (Sigma-Aldrich, St. Louis, MO, USA). The cultures were kept at 37 °C in a humidified atmosphere containing 5% CO_2_ (Water Jacketed CO_2_ Incubator, Thermo Scientific). The culture medium was replaced every 2–3 days, and at 80% confluence, cells were passaged using a trypsin–EDTA solution [0.05% (m/v) trypsin and 0.02% (m/v) EDTA.

##### 5.2.4.2 Toxicological Effects by Indirect Contact of Scaffolds and GSO on Cell Viability

GSO samples (0.6, 0.3, 0.15, 0.075, and 0.032%; v/v) and the developed scaffolds (CS, GSO3, CGSO6, dimensions of ∼1.5 cm^2^, previously sterilized by UV-radiation) were incubated in Ham-F-10 medium containing 10% FBS (2 mL) at 37 °C for 24 h. After, 100 μL of conditioned medium, supplemented with resazurin dye (7-hydroxy-3*H*-phenoxazin-3-one 10-oxide; Sigma-Aldrich, St. Louis, MO, USA) at 40 μM, was added on Vero cells, previously seeded into 96-well plates (Nest Biotechnology, Rahway, USA), at 1.0 × 10^4^ cells per well in sextuplicate. Then, cells were maintained for 24 h in a humidified atmosphere containing 5% CO_2_.

Three independent experiments were performed corresponding to independent scaffold preparation. The cytotoxic effects of GSO oil and scaffolds were determined by the staining of attached cells with crystal violet dye,^104^ and metabolic activity by resazurin reduction into resorufin.^56^ After the treatment, the fluorescence reading of the resazurin reduction in resorufin was performed in the microplate reader (BioTek Synergy HT Multi Mode Microplate Reader, Santa Clara, CA, USA), with 530 nm excitation and 590 nm emission. After, the medium was aspirated, and the cells were stained with crystal violet staining solution (0.5%), washed, and air-dried. Then, methanol (200 μL) was added to each well, and the absorbance was measured at 570 nm by using a SpectraMax reader (Molecular Devices, CA, USA). Data were obtained from three independent experiments in triplicate, expressed as the mean ± standard deviation (SD), and represent the percentage of cell viability by metabolism and integrity cell, calculated using the control group as 100%.

### 5.3 Statistical Analysis

All data are presented by mean ± SD of triplicate experiments. OriginLab was used to evaluate the statistical significance through one-way analysis of variance for between-groups comparisons, followed by a Tukey’s posthoc test for multiple comparisons or Tukey’s posthoc test to compare each of a number of treatments with a single control. Values of *p* < 0.05 were considered statistically significant.

## Supplementary Material



## Notes

Methodology for the apparent contact angle evolution test; apparent contact angle for the scaffolds (CS, CGSO3, and CGSO6) at different times (0, 4.5, and 8 s); and representative images of these scaffolds at the same time points (PDF)
