# Supplementary material for: Freeze-Dried Chitosan Scaffolds Containing Grape Seed Oil for Wound Healing Applications
Source: ACS Omega. 2025 Oct 6;10(41):48500–13. doi: 10.1021/acsomega.5c06168 (PMC12547589; doi:10.1021/acsomega.5c06168)
Supplement: Supplementary file 1 [file ao5c06168_si_001.pdf]

Supporting Information

Freeze-dried chitosan scaffolds containing grape seed oil for wound healing applications

Erik F. dos Santos [1] Larissa R. Lourenço [1], Carlos A. da Silva [2], Juliana Marchi [1*]

[1]Center of Natural and Human Sciences, Federal University of ABC, Santo André - SP, Brazil

[2]Center of Natural and Human Sciences, Federal University of ABC, São Bernardo do Campo - SP, Brazil

* Corresponding Author: Juliana Marchi (juliana.marchi@ufabc.edu.br)

Table of Content:

#	Content	Page
1	**Table S1**: Apparent contact angle of the scaffolds (CS, CGSO3, and CGSO6) at different times (0, 4.5 and 8 seconds).	S2
2	**Figure S1:** Representative images of the water droplet on the surface of the scaffolds (CS, CGSO3, and CGSO6) at 0, 4.5, and 8 seconds.	S3

Apparent contact angle evolution: The wettability of the surfaces of freeze-dried scaffolds (CS, CGSO3, and CGSO6) was investigated by measuring the apparent contact angle (θ_{ap}). The tests were performed under ambient conditions and at room temperature. A drop of distilled water (~40 µL) was dispensed onto the scaffolds using a contact angle goniometer (Phoenix MT, SEO, South Korea). Immediately after the deposition of the water droplet, successive images were captured every 100 ms for 8 s. To analyze the dynamics of droplet penetration into the scaffolds, three time points were used (t=0 s, t=4.5 s, and t=8 s). The apparent contact angle was measured using the ImageJ software. Measurements were performed in triplicate.

Table S1: Apparent contact angle of the scaffolds (CS, CGSO3, and CGSO6) at different times (0, 4.5 and 8 seconds).

Scaffolds	t=0 seconds	t=4.5 seconds	t=8 seconds
**CS**	77 ± 10	-	-
**CGSO3**	77 ± 12	75 ± 12	70 ± 15
**CGSO6**	72 ± 2	71 ± 2	60 ± 5

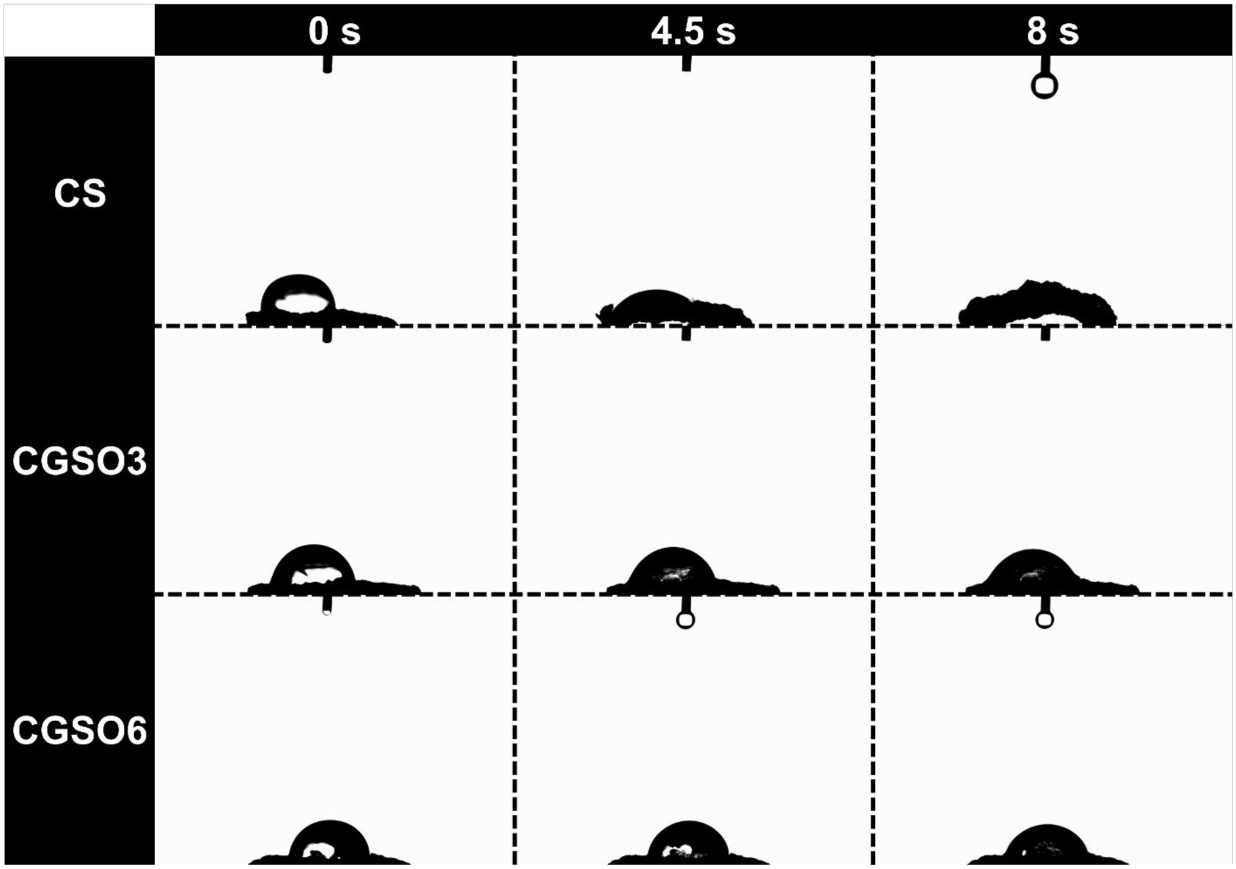

Figure S1: Representative images of the water droplet on the surface of the scaffolds (CS, CGSO3, and CGSO6) at 0, 4.5, and 8 seconds.
